# Introducing the nutrition & metabolism section of journal of translational medicine

**DOI:** 10.1186/1479-5876-10-17

**Published:** 2012-01-30

**Authors:** Laura Soldati, Elena Dogliotti, Irene Camera, Annalisa Terranegra

**Affiliations:** 1 Department of Medicine, Surgery and Dentistry, Università degli Studi di Milano, Milan, Italy

## Our food should be our medicine. Our medicine should be our food. Hippocrates (460 BC - 377 BC)

### 

Nutrition experts, as well as specialists of different pathologies, are disclosing an ever increasing interest for protocols devoted to the welfare of patients and to prevention of nutrition related diseases.

In fact, it is extensively acknowledged that nutrition plays a primary role in those complex diseases most widespread in the western countries like cardiovascular disease [1], cancer [2], Alzheimer's disease [3], diabetes [4], nephrolithiasis [5] etc. that account for over 60% of deaths world-wide.

Even in our country, Italy, and in other South Europe's countries, until now protected by the Mediterranean diet, which in 2010 was awarded by UNESCO the human heritage status, the next generation will be the first with a life expectancy lower than of their parents, principally due to unhealthy eating habits and the obesity epidemic.

To counteract this trend, in the last decade attention has been focused on the human nutrition field, with significant research topics being new molecules contrasting cholesterol accumulation in the arterial walls or capable of diminishing visceral fat [6], the role of bacterial microflora [7], the production of functional foods, studies on antioxidant properties of vitamins and polyphenols, etc.

Moreover, beside knowledge acquired with traditional investigation techniques, the field of human nutrition can now avail itself of the new omics techniques that could give a significant contribute to mapping the interactions between genetics, nutrition and health [8].

In the area of Translational Medicine, we believe that the field of nutrition ought to be taken into greater account, as an interaction and mutual validation agency between basic and applied research, in order to generate advanced applications for diagnosis and therapy and to develop at the same time new instruments of investigation.

We here propose a new section devoted to the rapid publication of research papers on all aspects of nutrition with applications in medicine and biotechnology.

This section welcomes contributions that employ traditional clinical, metabolic and epidemiologic methods, but is also open to molecular and cell biology research applied to animal and human nutrition.

Topics include: diet interactions in chronic diseases, gene-environment interactions, genes and cell metabolism, protein and aminoacid metabolism, lipid metabolism and diseases, carbohydrate metabolism and diseases, micronutrients and functional foods.

Other topics relevant to nutrition will be appreciated as well.

## References

[B1] Bhupathiraju SN Tucker KL Coronary heart disease prevention: nutrients, foods, and dietary patterns Clin Chim Acta 2011 412 1493 1514 10.1016/j.cca.2011.04.038 21575619 PMC5945285

[B2] Ross SA Evidence for the relationship between diet and cancer Exp Oncol 2010 32 137 142 21403607

[B3] Ramesh BN Rao TS Prakasam A Sambamurti K Rao KS Neuronutrition and Alzheimer's disease J Alzheimers Dis 2010 19 1123 1139 20308778 10.3233/JAD-2010-1312 PMC2931824

[B4] Mangou A Grammatikopoulou MG Mirkopolou D Sailer N Kotzamanidis C Tsigga M Associations between diet quality, health status and diabetic complications in patients with type 2 diabetes and comorbid obesity Endocrinol Nutr 2011 Epub ahead of print 10.1016/j.endonu.2011.10.003 22197574

[B5] Meschi T Schianchi T Ridolo E Adorni G Allegri F Guerra A Novarini A Borghi L Body weight, diet and water intake in preventing stone disease Urol Int 2004 72 suppl 1 29 33 15133330 10.1159/000076588

[B6] Last AR Ference JD Falleroni J Pharmacologic treatment of hyperlipidemia Am Fam Physician 2011 84 551 558 21888306

[B7] Scarpellini E Campanale M Leone D Purchiaroni F Vitale G Lauritano EC Gasbarrini A Gut microbiota and obesity Intern Emerg Med 2010 5 suppl 1 S53 56 20865475 10.1007/s11739-010-0450-1

[B8] Kussmann M Affolter M Proteomics at the center of nutrigenomics: comprehensive molecular understanding of dietary health effects Nutrition 2009 25 1085 1093 10.1016/j.nut.2009.05.022 19665868

